# Stress on local and international psychotherapists in the crisis region of Iraq

**DOI:** 10.1186/s12888-020-02508-0

**Published:** 2020-03-10

**Authors:** Jan Ilhan Kizilhan

**Affiliations:** 1 Institute for Psychotherapy and Psychotraumtology, University of Duhok, Zakho Street, Duhok, Iraq; 2 Institute of Transcultural Health Science State University Baden-Württemberg, Friedrich-Ebert-Strasse 30, D-78054 Villingen-Schwenningen, Germany; 3 Department of Transcultural Psychosomatic, MediClin Donaueschingen, Donaueschingen, Germany

**Keywords:** Psychotherapists, Conflict areas, Traumatization, Violence, Stress factors, Islamic state

## Abstract

**Background:**

Local and international Psychotherapists working with war survivors are confronted with a whole variety of burdens. The close contact to their clients and the conversations about terror, flight and genocides, they are frequently subject to vicarious traumatization resulting from these events and reveal a risk of developing secondary traumatization.

**Methods:**

We interviewed 54 local psychotherapists and 28 psychotherapists originating from abroad who were treating survivors of ISIS terror in refugee camps organised by non-government organisations (NGOs).

**Results:**

The results show that local and non-local psychotherapists who work in a context of humanitarian crises reveal a risk of developing secondary traumatization. The results of this present study would also indicate that the secondary traumatization depends both on individual characteristics such as bonding styles and personal experiences and also on the ambient characteristics such as the degree of traumatization of the patients.

**Conclusion:**

The study revealed that the local and non-local psychotherapists had a range of different pressure factors and helpful resources which indicated that better caring/support, supervision and organization are needed to enable psychotherapists to perform more effectively in war regions from the point of view of preventive healthcare.

## Background

In 2019, the United Nations refugee agency (UNHCR) registered 70.8 million people who had been forcefully displaced. Of these, 25.9 million people were classified as refugees who had been forced to leave their homeland on account of persecution, war or violence [1].

In 2014, people in Iraq and Syria, in particular, were subjected to considerable stressors due to “Islamic State” terror, when ISIS overran vast areas of Syria and Iraq [2]. They used severe brutality, especially towards long-established peoples, religious minorities in the country, particularly towards the Yazidis. Huge numbers of men were executed, thousands upon thousands of women and children were kidnapped and deliberately subjected to sexual violence [3]. This situation led to massive movements of refugees. The Duhok region, which borders with Turkey in the north and Syria in the south-west and which in 2015 had a population of approx. 1.5 million [4], took in the vast majority of the internally displaced persons. In the Duhok region alone there are currently (2019) 350,231 internally displaced persons and 21 refugee camps. Throughout Iraq there are 2,045,718 internally displaced persons in such camps [5].

Above all, it has been shown that the experience of violence and torture can increase the risk of developing a mental disorder, especially an affective disorder and a post-traumatic stress disorder [6, 7]. The life of many refugees is characterised by competition when looking for work, a difficult living situation in the camps, rejection in the homeland, the experience of sexual and physical violence and cultural uprooting. This in turn leads to conflicts in the social environment and feelings of helplessness, worry, insecurity and hopelessness [3, 8, 9]. Several studies have examined the prevalence rates of psychological disorders among refugees and internally displaced people in 21 countries [10]. These studies have found different prevalence rates of mental disorders. However, a systematic review points out that post-traumatic stress disorder (3–88%), depression (5–80%) and various forms of anxiety disorders (1–81%) are the most prevalent disorders. Merely 12 studies referred to other psychological disorders. The previously mentioned studies, a systematic review and other findings have drawn attention to a process in which traumata caused by human action (torture and predominantly sexual violence) precede the emergence of PTSD [10, 11] and co-morbid depression [12]. Moreover, these studies and the systematic review suggest that demographic and socio-economic factors shaping the environment of displaced groups, for example internal displacement, potentially influence psychological health significantly. For instance, the coincidence of living in a refugee camp, unresolved conflict issues, unpromising prospects and financial and material insecurity is frequently associated with reduced psychological health [11–13].

In northern Iraq, for example, there are currently merely 28 psychiatrists and 26 approbated psychotherapists, who work in three psychiatric institutions or for local non-governmental organizations, for 6 million people. Financial problems frequently represent an obstacle to psychiatric support. For instance, health insurance companies do not cover psychiatric out-patient support. Moreover, organizational difficulties hamper psychiatric support. For instance, difficult and precarious conditions in refugee camps and a poor infrastructure in towns and rural areas cause difficulties in providing psychiatric care. Finally, up to now the work of local psychotherapists and psychotherapists in general is not recognized as a profession [14].

According to WHO figures (2018) [5], there are 22 international and 8 national non-government organisations focusing on health care working in Iraq at the moment in addition to the meagre care structures mentioned above. Only nine of these are working in the Dohuk region. NGOs frequently work according to the MHPSS approach (Mental Health and Psychosocial Support). MHPSS describes all types of support, local or otherwise, which is geared to protecting or strengthening psycho-social welfare [15]. This includes consultations with trained lay people as well as with specialised psychotherapists. In 2018 relief agencies were able to take care of approx. 1.3 million people in need. In this context, up to now merely 10, 294 people out of 360,000 IDPs were provided with MHPSS. An insufficient health care structure, a low number of professional staff, little financial resources and other causes [16] make an adequate professional and nationwide psychotherapeutic treatment system impossible [14].

As a rule, local psychologists work in refugee camps for the local health authorities and the international NGOs. In addition, many psychotherapists from abroad work for the various NGOs in the refugee camps and in the few treatment centres. They are confronted with a range of different challenges in their daily work with traumatised persons [17]. As a result of their close contact with their patients and conversations about terror, flight and genocide, psychologists are frequently exposed to these traumatic events second-hand [18]. The ever-recurring exposure to the traumatic memories of their patients can result in the transfer of typical trauma symptoms such as hyperarousal, avoidance and intrusion on to the psychologist and psychotherapist, even if they have never been exposed to the traumatic events [19]. This phenomenon is known as secondary traumatization and has been identified in various medical and paramedical professions such as counselling psychologists, physiotherapists, doctors, nurses and trauma therapists [19–21].

Psychotherapists from abroad suffer from further disadvantages such as time contracts, minimal in-service and further training and supervision. As a rule, they are a minority profession in an NGO [14]. In addition to structural stressors such as location (camps, centres near the war zone) [22] the possible lack of specialisation of the psychotherapists, for example no additional training to become trauma therapists, plays an important part in the work in conflict areas [23].

At the same time local psychologists are deployed in these conflict areas who perhaps themselves have been victims of persecution or flight or who have been taken hostage. In addition to their work and possible traumatization they have to try to organise their lives long-term in these conflict areas [21, 24]. They have the advantages, of course, that they share the same language and culture as their patients, yet at the same time, given insufficient training, they cannot distance themselves professionally from them. In Iraq the profession of psychotherapist is unknown. Most of the psychologists who work with traumatised patients have had no training as psychotherapists and have acquired their knowledge through in-service training courses and seminars [14, 24]. Only since 2017 at Duhok University Institute of Psychotherapy and Psychotraumatology, thanks to support from Germany, have psychologists been trained to bachelor level as psychological psychotherapists as part of a master’s degree course [14, 24].

The general prevalence of pressures such as secondary traumatization when working in war zones and conflict areas underlines the need for research into predicators for pressures on specialists who work with severely traumatised patients. Yet, so far, there is not much data available, in particular as regards psychotherapists in conflict areas. Psychotherapists are often deployed as an expert committee for the diagnosis and treatment of (mental) suffering caused by war traumas rather than the object of research themselves [25, 26]. We have therefore decided, by means of the qualitative and quantitative collection of data, to carry out research into the psychological resources and stresses of psychotherapists in Iraq. From this, we hope to work out ways of supporting psychotherapists in conflict areas, so that they can process existing conflicts and possibly reduce their number.

## Methods

With the help of the Baden-Württemberg federal state government, an Institute of Psychotherapy and Psychotraumatology was opened in northern Iraq in 2017 under the auspices of Baden-Württemberg Cooperative State University and the University of Tübingen. A master’s course designed to train psychotherapists was offered, based on the German Psychotherapy Act, so that the patients could be treated by psychotherapists in situ. Long-term, it is envisaged that the psychotherapy will be integrated into the health care system there, something which is not the case to date, either in Syria or in Iraq [14, 24].

The psychologists who have been doing a psychotherapy course at the University of Duhok and who, at the same time, have been treating traumatised patients in the refugee camps under supervision since 2017 were the ideal group to form a random sample for collecting data. These students will graduate as qualified psychotherapists after 3 years in March 2020. A further group of psychotherapists were questioned who work from abroad for the various NGOs and centres in Duhok Province.

To qualify for inclusion, participants had to be qualified psychotherapists or who will be graduated in 2020 and who had worked for at least 2 years with traumatised patients in refugee camps. They had to have had at least 2 years’ experience in the job and had to volunteer for the study.

We were able to find 82 participants. All participants accepted the interviews. There were no rejections. A guideline interview was developed and then discussed with the interviewers, corrected and then accepted in the final version. The interviews for the qualitative part of the survey were recorded, transcribed and rendered anonymous by four trained psychologists.

The Institute for Psychotherapy and Psychotraumatology at University of Duhok which works in cooperation with local and international non-governmental organizations contacted the interviewers. Every psychologist contacted and asked by the author agreed to conduct the interview. After the study had been explained, written consent was obtained from all participants. The participants were visited in their workplace by the trained psychologist and did the interview face to face, prompt and recorded with the audio the interview.

The interviews were recorded on a digital audio and then pseudonymised, transcribed and analysed on a PC. The evaluation was done according to the qualitative content analysis after Mayring [27]. The evaluation was supported by Atlas.ti 5.2.12 (ScientificSoftware Development GmbH). Categories were developed inductively from the material in an interplay with the theory (research questions). We have analysed the interviews on the basis of a code system with the following steps: selection of units for analysis, definitions of the dimensions for structuring, definition of the characteristics and development of the code system, description of definitions, examples and rules for coding, coding, extraction of the codes, revision of the code system and further coding and interpretation and preparation of results. In order to meet the quality criteria of qualitative research, in particular intersubjective comprehensible [28], two Master-students from the Institute of Psychotherapy and Psychotraumatology at the University of Duhok developed main categories and sub-categories from the therapists’ responses inductively, independently of each other. Subsequently, the classifications were compared and discussed in the sense of a consensual coding. This procedure ensures both inter-subjectivity and comprehensibility of the results [21]. During consensus meetings, major categories and subcategories were discussed with the two Master-students until mutual agreement was reached. The author (JIK) recoded the material according to the final categories.

In this study, validated quantitative psychometric survey instruments as well as self-developed quantitative questionnaire-items were used. The ethics board of University of Duhok in Northern Iraq approved of this study.

For the quantitative part of the study the following test instruments were used:

### Sociodemographic

Sociodemographic data and context characteristics regarding the work and personal trauma or flight experiences were assessed.

### Questionnaire for secondary traumatization (QST)

The Questionnaire of Secondary Traumatization (FST) [19, 29] was used to assess the severity of secondary traumatization. The FST consisted of five secondary traumatization subscales: ‘intrusion’, ‘avoidance’, ‘hyperarousal’, ‘parapsychotic sense of threat’ and ‘PTSD-comorbidities’. Participants were prompted to rate how often these symptoms occurred during the first week of the time period in question with the highest level of distress in the professional context (here: psychotherapy with traumatized survivors of ISIS Terror). The FST showed a high internal consistency with α = .94 [29].

### Statistical analysis

As regards the sample description, we used means, percentages and distributions. For the analysis of differences in means, Mann-Whitney-U-tests for independent samples were applied, since the data were not normally distributed. To test correlations of determinants with the respective outcome (FST-scores), the Spearman rho test was applied as a nonparametric measure. Multiple linear regression analyses were performed to assess whether the determinants were associated with FST-scores and to identify potential risk and resilience factors. The level of significance for all analyses was set at α = .05. All statistical analyses were performed using IBM SPSS Statistics version 24.

## Results

Of the local therapists (*n* = 54), the mean age was 32 years, with a percentage of 61% men and 39% women. In the case of the psychotherapists (*n* = 28), who were working in Iraq from abroad, the percentages were 22% men and 78% women. Most of those questioned at the time of interview had at least a bachelor’s degree in psychology and in addition to 3 years’ job experience they had worked for at least 2 year’s with traumatised patients in Iraq. All of the psychologists from abroad (42%) stated they had obtained a master’s degree in psychology and that they had completed a psychotherapist training program. Moreover, they (30%) stated that they had gained work experience in other crisis and conflict areas, for example Rwanda, Jordan and Bosnia.

This study did not show any significant difference between male and female interviewees. This applies to the entire area of this study.

### Prevalence of secondary traumatization

Applying the QST diagnostic criteria, secondary traumatization was present in 44.4% of the participating local psychotherapists with 28.6% of the psychotherapists from abroad showing severe secondary traumatization and 18.5% of the local and 14.3% of the psychotherapists from abroad indicating a moderate secondary traumatization (Table 1). The mean secondary traumatization score was 58.74 (*SD* = 14.62).

**Table 1:** Secondary traumatization of psychotherapists and personal trauma

QST	No ST N (%)	Moderate ST N (%)	Severe ST N (%)	Personal Trauma (Flight, War, Violation, etc.) N (%)
Whole Sample	36 (43.9)	14 (17.0)	32 (39.0)	17 (20.7)
Local Psychotherapists	20 (37.0)	10 (18.5)	24 (44.4)	12 (22.2)
Psychotherapists from abroad	16 (57.1)	4 (14.3)	8 (28.6)	5 (17.9)

*ST* Secondary traumatization, *QST* Questionnaire for secondary traumatization

A personal trauma history was reported by 22.2% of local psychotherapists and by 17.9% of the psychotherapists from abroad. 44% of the local psychotherapists and 20% of the psychotherapists from abroad had experienced at least one traumatic event in their life.

A personal history of flight was reported by 18.5% of the local psychotherapists.

No psychotherapist from abroad had experienced a personal history flight.

### Resources and stresses

#### Psychotherapists from abroad

The qualitative analysis of the data revealed various factors which could be identified as both stressors and resources.

Identification with the job (75%) and relation to other members of staff of the organisation in Iraq (71%) is seen as a resource, but which is sometimes accompanied by conflicts. Belonging to a team and being able to help people via their job (53%) is, on the whole, seen as an important resource (Table 2).

**Table 2:** Resources and Stressors of the Therapists

	Resource	n	%		Stress Factor	n	%
	Therapists from abroad	n = 28				n = 28	
1.	Identification with the job	21	75%	1.	Not enough humanitarian and medical support in the refugee camps	22	79%
2.	Relation to NGO staff for whom they work	20	71%	2.	Therapy with survivors	22	79%
3.	Family, partnership, children	16	57%	3.	Insufficient financial remuneration	17	61%
4.	Help by means of psychotherapy	15	53%	4.	Lack of professional recognition/respect from the organisation they work for.	18	64%
5.	Professional and personal development	15	53%	5.	Distance from family and friends	17	61%
6.	Help victims of sexualised violence	8	29%	6.	Lack of a personal retreat	17	61%
7.	Get to know foreign people and cultures	12	43%	7.	Tense security situation	13	46%
	Local Therapists	n = 54				n = 54	
1.	Family and friends	49	91%	1.	Locals know only little about mental illnesses	46	85%
2.	To be able to help the victims of terror	45	83%	2.	Profession of psychotherapist is not recognised	42	78%
3.	Appreciation/respect in the community	35	65%	3.	Insufficient financial remuneration	32	59%
4.	Training and continuing medical education in psychotherapy	32	59%	4.	Threat from ISIS and other extremist groups	23	54%
5.	Contact with other psychotherapists	19	35%	5.	Concern for family members fighting against terrorism	16	30%
6.	Contact with foreign psychotherapists	19	35%	6.	No positive and political perspectives	16	30%
7.	They have work	13	24%	7.	Increasing radicalisation	12	22%

As the main stressors they see the work with severely traumatised people, their stories, and a certain helplessness, since they themselves can do little to help with factors such as nutrition, safety, political perspective, injustice actually experienced and the human rights situation in Iraq. They see the insufficient humanitarian and medical aid in the refugee camps as a great burden on themselves (79%).

Even the experiences and narrations of the children and young girls, such as rape, enslavement and torture, stay in their mind after the therapy session has finished (69%). The patients’ complaints such as high levels of emotionality, physical complaints, frequent dissociative attacks, a strong desire for medication and a desire to leave Iraq are a burden on both foreign and local psychotherapists.

The psychotherapists (62%) from abroad sometimes report communication problems because they do not always have good interpreters. As a result, they cannot classify the symptoms and their manifestation very well and cannot understand fully the role of women in patriarchal societies and the marriage rules in Iraq as well as the low esteem attached to psychological work in the community and with the authorities. But, on the whole, the psychotherapists from abroad regard themselves as competent (84%) and believe that they can help the people.

The psychotherapists originating from abroad were dissatisfied with the planning and duration of therapies, with the lack of transparency, the insufficient financial reward (79%) and the lack of personal respect (64%) from the organisation which employed them. The persons concerned were normally able to accept sudden changes in plan if they were handled transparently and with reasons. However, almost two-thirds of those questioned said that sudden changes in plan reinforced their impression that the organisation was unreliable.

The negative aspects of such a close social grouping – a combination of being in a group which had been artificially thrown together and the impression of a lack of personal support (61%) – hardly detract from the advantages. However, this functioning of a feeling of togetherness and solidarity was also the result of the external threatening situation (46%), the high work load and from living in a foreign country, despite the heterogeneity of the team members.

Both female and male therapists from abroad see the great distance from their families, children or friends as a stress factor 43% (*n* = 12). As a result, they are not able to help with problems of their partners or families. The regular absence of a member of the family inevitably leads to a role change and this often results in the loss of family obligations, a loss of the right to have a say in things and an identity crisis on the part of the person returning home. This, in the case of a psychologist, together with the different experiences resulting from a job full of suspense in Iraq and the everyday existence of the partner at home often generates problems of understanding, communication and recognition. There are plenty of means of communication which can bridge this spatial and emotional distance such as telephone and Internet, yet in the opinion of more than three quarters of those interviewed these cannot compensate for the lack of personal contact.

#### Local therapists

The local therapists grew up in the country, speak the language of the patients and live there with their families. They see the family as their biggest resource (91%). The social network of the local female and male psychologists consists of the core family and the immediate circle of friends and acquaintances; the emotional support, but also the potential for conflict which arises out of this, make the social background, double edged, into a significant resource but also a stress factor while the political situation in Iraq is not resolved and while, in places to the end of 2015, ISIS was fighting 30 to 50 km away against Kurdish and Iraqi soldiers. Some of the female psychologists questioned (30%) were still directly affected by the ISIS fighting, because their husbands were fighting as Kurdish Peshmerga at the border with Syria.

In general, the local therapists are highly motivated to help their traumatised compatriots (71%) and see the therapeutic work, despite the stress, as an important resource. However, since the profession of psychotherapist is unknown in Iraq, they often have to fight for recognition by the authorities (78%) and they see the poor remuneration as an additional stressor (59%). They are however also very motivated to receive in-service training in the field of psychotherapy (59%) and they see the contact to the therapists from abroad as an enrichment, since they can learn a lot from them.

Local psychologists do not regard themselves as sufficiently qualified (59%), but they have to do the work since there are no specialists. They would like additional help in the treatment of mentally ill people. They are positive about the help which they get from the NGOs in the shape of additional specialist training, but they say it is insufficient from a culturally-aware point of view.

Other stress factors which local as well as non-local therapists perceive are connected to difficult conditions regarding the transport system in refugee camps (local therapist (LT) 42%/ non-local therapists (NLT) 46%) as well as the insufficiently arranged and furnished rooms for individual and group therapy (LT:54%, NLT:57%) and to therapists’ impression that they cannot cooperate with physicians as closely as they wish to (LT:54%, NLT:62%). They are under the impression that their work is not sufficiently acknowledged. Moreover, they express concerns with regards to the psychiatrists who, in their point of view, hand out huge amounts of medication too prematurely (LT: 38%, NLT:52%).

The accommodation of the therapists from abroad is regarded as insufficient. There is no constant power supply and having to share accommodation with several people restricts their privacy (56%). Recreational activities are restricted and there are only few opportunities to pursue sporting activities, for example (42%). The weather too, over 40 degrees in summer and minus temperatures in winter, leads to stress and adaptive difficulties (34%).

The therapists from abroad who were asked had observed some therapists reverting to escape mechanisms, for example dissolute enjoyment of pleasure (sex, nicotine, alcohol) and this they judged negatively (36%). The local therapists cited eating too much, too little exercise and a lot of television (44%).

Therapists said that the paltry support from the population was additionally stressful: they thought the population were partly disinterested and uninformed and partly manipulated by the media. They could understand and appreciate this because of their own doubts, but such a situation only reinforced their misgivings. In addition to the lack of public appreciation, there was an absence of financial reward.

## Discussion

This study examined the stressors and resources of local therapists and those from abroad working with survivors of ISIS terror in refugee camps in northern Iraq. The study showed that working with patients with a trauma disorder who have suffered physical, mental and sexual violence as a result of being taken hostage by ISIS, can lead to the trauma symptoms being transferred to the psychotherapists treating them. 44.4% of the local psychotherapists and 28.6% of psychotherapists from abroad showed signs of severe secondary traumatization: 18.5% of the locals and 14.3% of the psychotherapists from abroad displayed moderate secondary traumatization. These rates of prevalence show a considerable mental health risk for psychotherapists working with traumatised survivors in the crisis area in Iraq. From a statistical point of view, psychotherapists working with the severely traumatised and survivors of ISIS terror (captivity, torture, rape, group persecution, compulsory conversion etc.), themselves display trauma symptoms more often [23, 30]. This high degree of secondary traumatization is of course critical, but not completely surprising.

Psychotherapists from the Kurdistan region of Iraq (39.0%) have themselves experienced at least one traumatic incident in their lives [31, 32]. This life-time prevalence rate is higher than the rate of 23.8% for traumatic experiences in the general population in Germany [31]. Local psychotherapists themselves were witness to, or in part directly or indirectly victims of ISIS terror, and were afraid that their region too could be occupied by the terror organisation [14]. There are also indications that people working in crisis and post-conflict areas are more likely to have had a traumatic experience than the general population [21, 33]. Therefore, a person’s own trauma history is a highly relevant risk factor for secondary traumatization than it is in the case of local psychotherapists. In other studies, it has been found that, when psychotherapists have undergone similar traumata to their patients, this is a reason for a lack of necessary distance and at the same time can lead to secondary traumatization [10, 19, 32–34]. The results of our study seem to confirm this assumption.

The qualitative part of the study was able to demonstrate that positive and negative influencing factors such as social environment, family, contacts, communication and insufficient humanitarian help in Iraq play a vital role. As positive resources, psychotherapists from abroad experience the identification with their profession and that as psychotherapists they are able to help people and be a support to partnership and family. Important resources for the local psychotherapists first and foremost are their family, the fact that they are “helping people” and the appreciation they get in the community. The negative factors for the psychotherapists are the insufficient humanitarian aid throughout the whole country, the therapy with survivors, insufficient financial remuneration and recognition from the organisation they work for.

For local psychotherapists, the disadvantages are their fellow countrymen’s meagre knowledge of mental illness, the lack of appreciation shown to the profession of psychotherapist, the threat from extremists and worries about their families.

Psychotherapists from abroad live in “forced communities” without any private space, a situation which is not always free of conflict. The high number of traumatised refugees and the “horror stories” they hear also makes them doubt the effectiveness of their psychotherapeutic activity. The people need tents, food, and to be able to grieve for the members of their families who have been killed or who are being held in captivity and with this stressor in mind “therapy is difficult, sometimes impossible.”

The results of this study confirm that the increased traumatising potential in the everyday work of the psychotherapist must not be underestimated [33, 34]. The region where the psychotherapists are working is safe to some degree but there is no guarantee that this will be the case the following day. Contact to the patients who report about their experiencing violence and flight also leads to increased stress and sometimes to a feeling of helplessness in the face of infinite violence, above all for those who have had to experience violence towards women and children [18, 34]. In addition, the poor infrastructure, long transport routes and unfamiliar climatic and hygiene conditions make it difficult to establish a working routine. This latter is sometimes due to language problems or to working with interpreters and the fact that they have to do the therapy in tents or simply outside in the camp. The discrepancy between the psychotherapists’ high trauma load and the few concessions made by an emotional viewpoint contingent upon this would seem to be mere social expediency [18]. High avoidance levels seem to be evident, namely that they play down their mental stress; they are “professionals” and as such are immune to mental decompensation [34–38]. In the case of potential stigmatising topic areas such as psychological instability and social crisis situations, there is an automatized reversal of roles into that of the expert; even barely socially-acceptable coping strategies such as heavy smoking, alcohol or sexual debauchery have only been observed at a distance in other psychotherapists and are not confirmed from their own experience.

## Conclusions

The qualitative approach in this study has produced both a wide spread of information on the situation of local psychotherapists and those from abroad in Iraq and their current predominant working conditions and insights into their complex causal relationships and background. This suggests a need to take a closer look at working conditions and the aftercare of therapists and to develop models for better prevention and care. Especially due to increasing political conflicts and the increased use of psychotherapists in crisis areas, there is a need for more studies in this area. Through psychotherapeutic training in Iraq, which does not yet exist in health care systems, processes such as psychotherapeutic studies, accreditation of this profession and advanced training could facilitate the work of psychotherapists in this country.

## Data Availability

The data and materials of study are available from the corresponding author on reasonable request.

## Abbreviations

FST: Questionnaire of Secondary Traumatization
ISIS: “Islamic State”
MHPSS: Mental Health and Psychosocial Support
NGO: Non Government Organisation
PTSD: Posttraumatic Stress Disorder
QST: Questionnaire for secondary traumatization
UNHCR: United Nations refugee agency
WHO: World Health Organisation
